# Interannual and Spatial Variability in Maturity of Walleye Pollock *Gadus chalcogrammus* and Implications for Spawning Stock Biomass Estimates in the Gulf of Alaska

**DOI:** 10.1371/journal.pone.0164797

**Published:** 2016-10-13

**Authors:** Benjamin C. Williams, Gordon H. Kruse, Martin W. Dorn

**Affiliations:** 1 College of Fisheries and Ocean Sciences, University of Alaska Fairbanks, Juneau, Alaska, United States of America; 2 NOAA National Marine Fisheries Service, Alaska Fisheries Science Center, Seattle, Washington, United States of America; Sveriges lantbruksuniversitet, SWEDEN

## Abstract

Catch quotas for walleye pollock *Gadus chalcogrammus*, the dominant species in the groundfish fishery off Alaska, are set by applying harvest control rules to annual estimates of spawning stock biomass (SSB) from age-structured stock assessments. Adult walleye pollock abundance and maturity status have been monitored in early spring in Shelikof Strait in the Gulf of Alaska for almost three decades. The sampling strategy for maturity status is largely characterized as targeted, albeit opportunistic, sampling of trawl tows made during hydroacoustic surveys. Trawl sampling during pre-spawning biomass surveys, which do not adequately account for spatial patterns in the distribution of immature and mature fish, can bias estimated maturity ogives from which SSB is calculated. Utilizing these maturity data, we developed mixed-effects generalized additive models to examine spatial and temporal patterns in walleye pollock maturity and the influence of these patterns on estimates of SSB. Current stock assessment practice is to estimate SSB as the product of annual estimates of numbers at age, weight at age, and mean maturity at age for 1983-present. In practice, we found this strategy to be conservative for a time period from 2003–2013 as, on average, it underestimates SSB by a 4.7 to 11.9% difference when compared to our estimates of SSB that account for spatial structure or both temporal and spatial structure. Inclusion of spatially explicit information for walleye pollock maturity has implications for understanding stock reproductive biology and thus the setting of sustainable harvest rates used to manage this valuable fishery.

## Introduction

Ontogenesis and phenology are unique for a particular fish stock and often vary over time due to ecological and fishery effects. Gaining comprehensive knowledge about stock life history parameters (e.g., fecundity, maturity, age-structure) is expensive; therefore, fishery scientists endeavor to acquire as much information as possible during fishery-independent surveys to inform stock assessment models. However, the sampling methods chosen to characterize a stock, while perhaps maximizing the quantity of data obtained, may affect precision and accuracy of population statistics, leading to a biased view of the true status of a stock. Although some of these biases, such as gear selectivity, can be addressed by parameter estimation within stock assessment models, other biases, such as misspecified maturity ogives, require independent estimation by field studies [1, 2].

Marine fisheries management is often based on biological reference points (e.g., harvest rates or biomass levels) that specify the framework for sustainable harvest levels. These reference points are predicated on an assumed relationship between stock reproductive potential (RP) and subsequent recruitment. Contemporary age-structured assessment models generally use an estimate of spawning stock biomass (SSB), i.e., the biomass of female spawning fish, to approximate stock RP. This approximation inherently assumes a proportional relationship between SSB and RP [3, 4]. Maturity at age or length is a key aspect of reproductive biology that is central to estimating both RP and SSB. Bias in stock parameters defining maturity ogives can lead to fishery management decisions based on misspecified biological reference points. Additionally, these parameters may vary with fish density or environmental conditions. Functional relationships are often elusive, hindering our ability to properly incorporate population dynamics into stock assessments, including forecasts of stock responses to alternative management strategies.

Walleye pollock *Gadus chalcogrammus* (hereafter, pollock) is a moderately long-lived species (maximum age of 22 yr) that is widely dispersed throughout the North Pacific Ocean [5]. The pollock fishery off Alaska is the largest fishery in North America, on the order of 1.3 million metric tons worth $343 million in exvessel revenue annually [6]. The volume of pollock harvested in the eastern Bering Sea fishery is an order of magnitude larger than that of the Gulf of Alaska (GOA). Nevertheless pollock comprises the largest portion of the groundfish catch (41% in 2013) by weight in the GOA [7]. There is some evidence that pollock spawning populations in the northern portion of the GOA are genetically distinct from pollock in Shelikof Strait [8], however uncertainty remains and pollock are managed with a statistical age-structured assessment model as a single stock in the central and western GOA [9, 10]. Maturity is incorporated into the stock assessment as the average maturity at age for the time period of 1983 to present, and does not attempt to track temporal variability or spatial trends in maturity [11].

**Fig 1:**
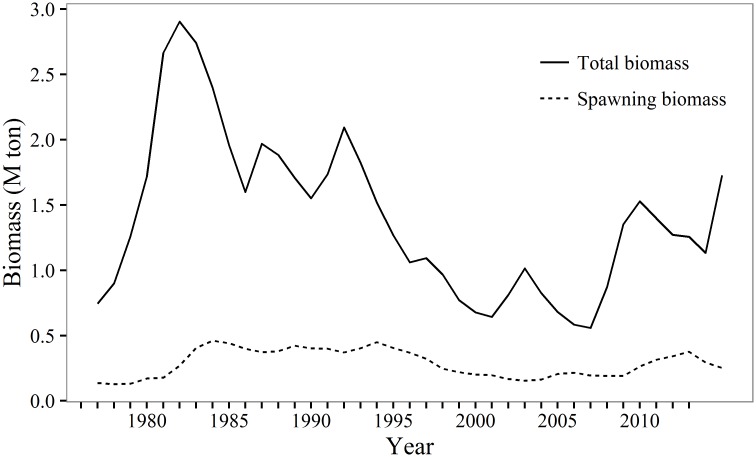
Walleye pollock biomass. Estimates of age 3+ total biomass (dashed line) and spawning stock biomass (solid line) of walleye pollock in the Gulf of Alaska (from Dorn et al. 2013).

**Fig 2:**
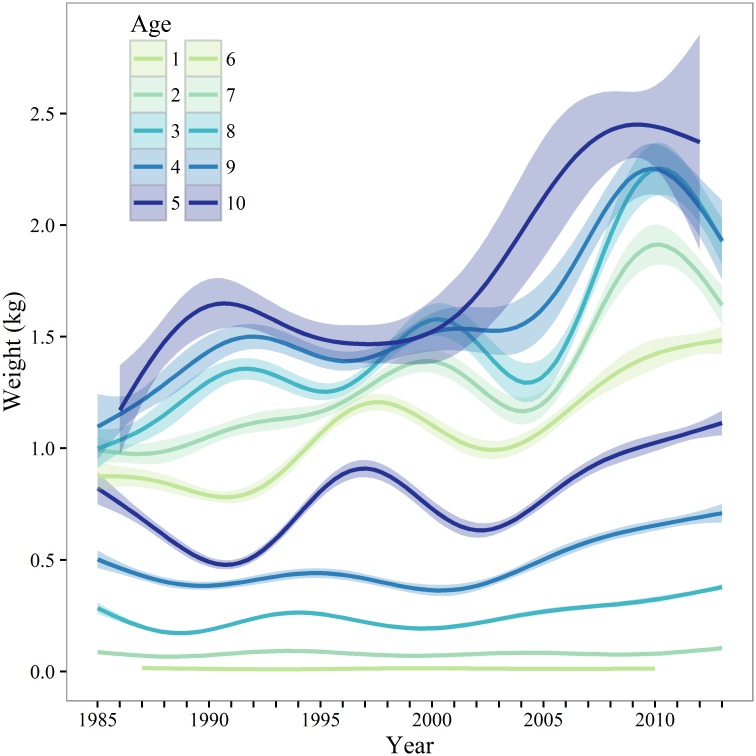
Walleye pollock weight at age. Female walleye pollock weight at age for ages 1–10 from fishery-independent samples in Shelikof Strait, Gulf of Alaska. Solid lines are generalized additive model estimates; shaded areas are 95% confidence intervals.

Length at 50% maturity (*L*_50_) for eastern Bering Sea pollock appears to be related to length at age, and there is some evidence for a density-dependent relationship between *L*_50_ and stock biomass [12]. In other species, maturity or total egg production can vary in relation to fish length and condition [4, 13, 14], the quality and availability of food resources, environmental conditions (e.g., temperature), and as a response to changes in stock biomass and fishing pressure [3, 15]. Spatial variability in GOA pollock maturity and potential relationships with these density-dependent and -independent factors are unknown, and temporal variability has been infrequently examined. Pollock growth and GOA stock biomass are inversely related (Fig 1) and weight-at-age has increased dramatically over 2000–2013, particularly for female pollock older than age-4 (Fig 2). However this relationship is less clear over 2007–2013, when the population numbers have increased and weight at age has remained high. Additionally, the GOA experiences significant environmental variability, including periodic climate regime shifts [16]. There are many potential effects of varying temperatures on pollock; for example, cool conditions appear to be associated with improved reproductive success and increase the abundance of the euphausiid *Thysanoessa* spp., an important prey of pollock [17]. Changes in individual pollock growth rates, perhaps associated with shifts in pollock biomass and environmentally driven changes in prey, may influence maturation rates and distribution of mature pollock in the GOA.

Trawl catches collected during annual Alaska Fisheries Science Center (AFSC) hydroacoustic surveys [18] provide samples to assess maturity of pollock in the GOA. The acoustic surveys were conducted along transects in Shelikof Strait, near Kodiak Island, Alaska, typically in March each year to estimate pollock biomass by location. Periodic trawl tows were utilized to examine the age and size structure of pollock schools observed via acoustics and to determine the species compositions [18]. Pollock were also sampled from these tows for maturity assessment, although sample sizes were not scaled to localized biomass. Macroscopic maturity estimates were determined using a 5-stage key [19] or an 8-stage key from 1996–2007 [20]. Maturity ogives were calculated from all samples during a given survey without regard to spatial variability. Ideally, to be representative, a maturity ogive for a population should be based on samples weighted by the relative biomass across the full geographic distribution of the stock [2].

The goal of our study was to identify annual and spatial patterns in GOA pollock maturity based on samples collected during NMFS acoustic surveys in Shelikof Strait during 1983–2013. In particular, we examined spatial bias in the estimation of a pollock maturity ogives and the influence of observed bias on estimates of SSB. While the pollock population is dispersed throughout the GOA, we chose Shelikof Strait only, as it contains the largest spawning concentration of pollock in the GOA, and it has been the most consistently sampled during annual assessment surveys [11].

## Materials and Methods

**Fig 3:**
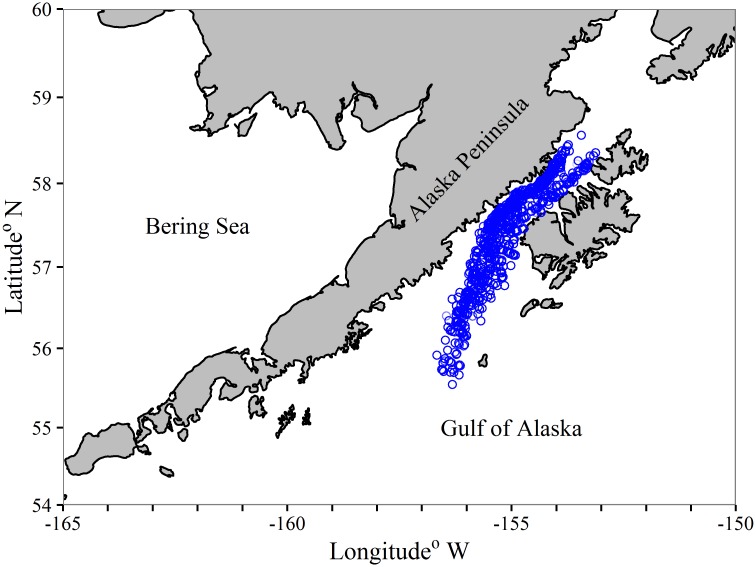
Map of walleye pollock sampling locations. Sample locations of walleye pollock used for maturity assessment over 1983–2013 in Shelikof Strait, Gulf of Alaska.

We analyzed data on gonad maturity of female pollock collected during annual acoustic surveys conducted by NMFS in the western GOA in February-March from 1983 through 2013. Maturity was estimated macroscopically using a 5-stage key developed for pollock [19]. This key was expanded into an 8-stage key in 1996 that was in use until 2007 [20], after which the 5-stage key was again employed. For our study, the maturity keys were reduced to a two-stage scale (mature/immature) with fish in pre-spawning, spawning, and spent stages classified as mature and fish in immature and developing stages classified as immature. Fork lengths (FL) were rounded to the nearest cm for each sampled fish and ages were determined by the AFSC Age and Growth Program [21]. Fish of age-10 and greater were binned as a plus group. Our analyses were restricted to pollock sampled from Shelikof Strait and to the southwest toward 55°30’ N, 157°W, west of Chirikof Island, as it is the region in the GOA with the most continuous sampling for pollock abundance and maturity (Fig 3) No maturity samples are available for 1999 and 2011 because surveys were not conducted in those years (Table 1). A total of 17,236 fish were available for age-based modeling and 34,342 fish for length-based modeling (Table 1). Associated length information was available for all fish that were aged, therefore the data used for age-based modeling was also used as an “equivalent dataset” for examining the explanatory power of age- and length-based models.

**Table 1:** Annual maturity sample sizes collected by length and age for walleye pollock in Shelikof Strait for 1983 to 2013.

Year	Length	Age
1983	2,394	1,103
1984	2,889	1,467
1985	2,091	1,183
1986	1,178	618
1987	733	643
1988	949	464
1989	1,102	545
1990	1,740	1,117
1991	675	567
1992	1,161	765
1993	1,365	624
1994	2,940	632
1995	1,243	575
1996	2,198	775
1997	1,547	853
1998	1,282	784
2000	1,294	363
2001	1,399	378
2002	667	326
2003	775	321
2004	712	440
2005	483	335
2006	691	487
2007	453	320
2008	426	248
2009	430	301
2010	462	244
2012	523	372
2013	530	386
Sum	34,332	17,236

Maturity *M* was modeled as a binomial response with a logit link using generalized additive models (GAMs) for the full 1983–2013 dataset to examine spatial and temporal variability. Explanatory variables included year, latitude, longitude and either age or length. The full GAM has form:

$$M=f_{1}\left(Age\right)+f_{2}\left(Longitude,Latitude\right)+f_{3}\left(Haul,bs=re\right)+Year+\epsilon ,$$ (1)

where the *f*’s are functions. To limit the analysis to biologically reasonable relationships the number of knots *k* or maximum degrees of freedom for the smoothing term applied to age or length, was restricted to 4 [22, 23]. Year was modeled as a categorical variable. Multiple maturity samples were collected from a given trawl haul *Haul* violating assumptions of independence; therefore, *Haul* was included as a random effect using the *bs = re* statement. All models were evaluated with the mgcv package in R version 3.1.2 [24, 25]. Final parameter estimates were calculated using restricted maximum likelihood. The relative explanatory power of predicting maturity by length or age, as well as reduced models, was examined using the Akaike Information Criterion [26, 27]. Comparisons between age- and length-based models were estimated on an equivalent dataset. Normal approximate standard errors were estimated in the mgcv package [25] on the predictor scale and transformed to the response scale.

Estimates of pollock biomass at length for 0.5 nmi transect segments (Provided by T. Honkalehto, NMFS, Seattle, pers. comm.) were used as prior weights on maturity data to generate a biomass-weighted maturity curve [2]. These biomass estimates were calculated by combining acoustic backscatter information along transects with size-composition data from associated trawl samples [28]. Although acoustic biomass estimates are produced for the continuous survey transects, samples for maturity estimation are from individual haul locations. Therefore, direct weighting cannot be applied due to the differing spatial extent of the two datasets. Instead, a classification and regression tree (CART) model [24, 29, 30] was implemented to identify regions with similar maturities over which pollock biomass could be summed.

Classification and regression trees are machine-learning procedures that recursively partition the data space based upon the ability of explanatory variables to predict the response variable [31]. In our application, the CART model provides regions *Region* with similar probabilities of a female fish being mature using the following model structure:

$$M_{Region}=Longitude+Latitude.$$ (2)

The CART model was constrained by a complexity parameter set to 0.01 that defined a level of model fit below which models were dropped via 10 fold cross-validation [30].

Prior weights for the maturity data were calculated by binning transect biomass data into 5 cm length increments (length group) by year and region. Estimates of transect biomass were assumed to be split 50:50 among males and females. Transect biomass estimates were length-structured rather than age-structured, however estimates of maturity by age are necessary for incorporation into an age-structured stock assessment. Therefore predicted age distributions by length group, region, and year were assigned using a conversion matrix (i.e., length-age key). This conversion matrix allows for a proportional allocation of ages to the spatial transect data through the designation of biomass at age for each region. Comparisons using biomass as prior weights were restricted to years with available data, i.e., 2003 through 2013 (excluding 2011). The reciprocal values of summed biomass by age, length group, year and region were used as prior weights in the top GAM models previously chosen using the AIC.

To generate comparable estimates for examining the influence of annual and spatial variability, maturity estimates for Shelikof Strait were also calculated via a generalized linear model (GLM; [24]), which is the method utilized in the stock assessment [11]. This estimate of maturity (base model) was used for comparison to the GAMs that explicitly incorporate spatial variability (spatial model) and the biomass-weighted spatial model (weighted spatial model). Graphical comparisons of averaged estimates from these models include 95% bootstrapped confidence limits [32]. The median size and age at maturity (*L*_50_ and *A*_50_, respectively) were estimated for each model with confidence limits calculated as two times the model-generated standard error.

Annual estimates of the numbers of pollock at age were obtained from the GOA age-structured stock assessment [Table 1.17 in [11]]. These estimates were generated via an age-structured stock assessment developed using AD Model Builder [33] that incorporates both fishery dependent and independent data sources such as fishery age and length catch compositions and NMFS trawl survey age and length catch compositions [11]. Annual pollock weight at age used in the stock assessment to calculate spawning biomass is based on Shelikof Strait survey data, and is considered to represent weights at time of spawning [11]. Female spawning stock biomass was calculated for a given year *i* as:

$$SSB_{i}=\sum W_{a,i}*M_{a}*N_{a,i},$$ (3)

where *W_a,i_* is the mean fish weight at age *a* in year *i*, *M_a_* is the average proportion of mature females (provided by the base, spatial, or weighted spatial models) at age, and *N_a,i_* is the number of fish of a given age in year *i* from the stock assessment [11]. Prediction intervals for SSB were estimated as two times the maturity model(s) estimated standard errors.

## Results

The inclusion of sample location improved model fits when compared to the base model (Table 2) for both length-based and age-based models. Variants of the global age or length models with terms removed were not considered an improvement because their AIC model weights were less than 1%, therefore they were excluded from further consideration (Table 2). Maturity at length was found to be a better descriptor of pollock maturity than maturity at age (Table 3), when compared using an equivalent dataset.

**Table 2:** Age- and length-based model fits with AIC values and AIC weights. Note that the age-based models were estimated on a reduced dataset and are not directly comparable to length-based models. The base model is identified with *, the spatial model (unweighted and weighted) is identified with †. Models are ranked from best to worst fitting. Where *age* = is the numeric fish age, *length* = individual fish length, *lon* = longitude in decimal degrees, *lat* = latitude in decimal degrees, *year* = the year sampling occurred as a factor, *haul* = individual hauls that samples originated from, incorporated as random effects, *(e)df* = model estimated degrees of freedom, AIC = Akaike information criterion, Δ_*i*_ = AIC difference, AIC weights is the relative likelihood of a model, *f* = smooth terms. Age and length models were fit on different datasets as more length data are available.

Model	(e)df	Deviance explained	AIC	Δ_*i*_	AIC weights
**Age-based models**					
^†^ *f*(age) + *f*(lon, lat) + *f*(haul) + year	358.29	75%	6,543	0	100.0%
*f*(age) + *f*(haul) + year	435.17	75%	6,661	118	0.0%
*f*(age) + *f*(lon, lat) + year	48.95	69%	7,397	854	0.0%
*age × year	58	62%	9,064	2,521	0.0%
*f*(age) + year	31.97	62%	9,079	2,537	0.0%
**Length-based models**					
^†^ *f*(length) + *f*(lon, lat) + *f*(haul) + year	461.24	77%	11,997	0	100.0%
*f*(length) + *f*(haul) + year	539.32	77%	12,106	109	0.0%
*f*(length) + *f*(lon, lat) + year	49.75	71%	13,999	2,002	0.0%
*length × year	58	67%	15,825	3,828	0.0%
*f*(length) + year	31.915	66%	16,403	4,406	0.0%

**Table 3:** The best age-based and length-based models (see Table 2) evaluated using an equivalent dataset to allow for model comparisons. Where *age* = the numeric fish age, *length* = individual fish length, *lon* = longitude in decimal degrees, *lat* = latitude in decimal degrees, *year* = the year sampling occurred as a factor, *haul* = individual hauls that samples originated from, incorporated as a random effect, *(e)df* = model estimated degrees of freedom, AIC = Akaike information criterion, Δ_*i*_ = AIC difference, AIC weights is the relative likelihood of a model, *f* = smooth terms. Age and length models were fit on different datasets as more length data are available.

Model	(e)df	Deviance explained	AIC	Δ_*i*_	AIC weights
f(length) + f(lon, lat) + f(haul) + year	348.87	76%	6,386	0	100.0%
f(age) + f(lon, lat) + f(haul) + year	358.29	75%	6,543	157	0.0%

**Fig 4:**
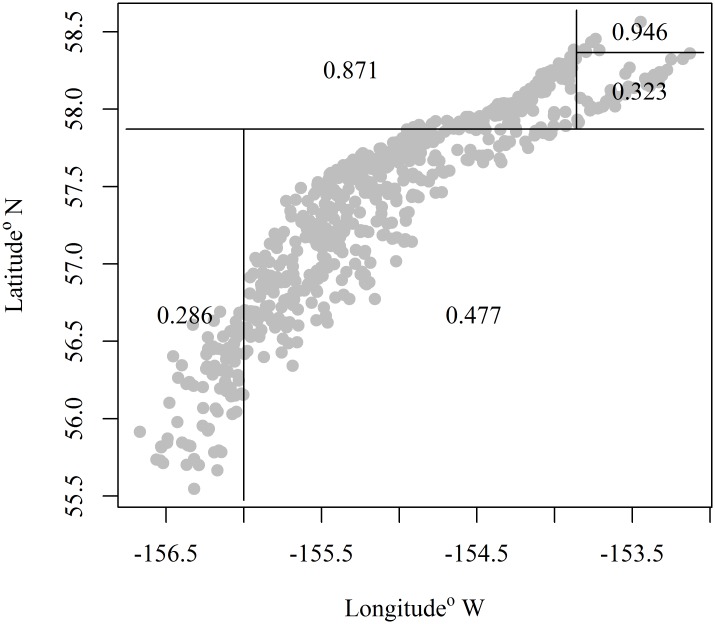
Classification regression tree. Proportion of female walleye pollock mature by area from a classification and regression tree model for the Gulf of Alaska. Numbers indicate the proportion mature in a given area.

**Fig 5:**
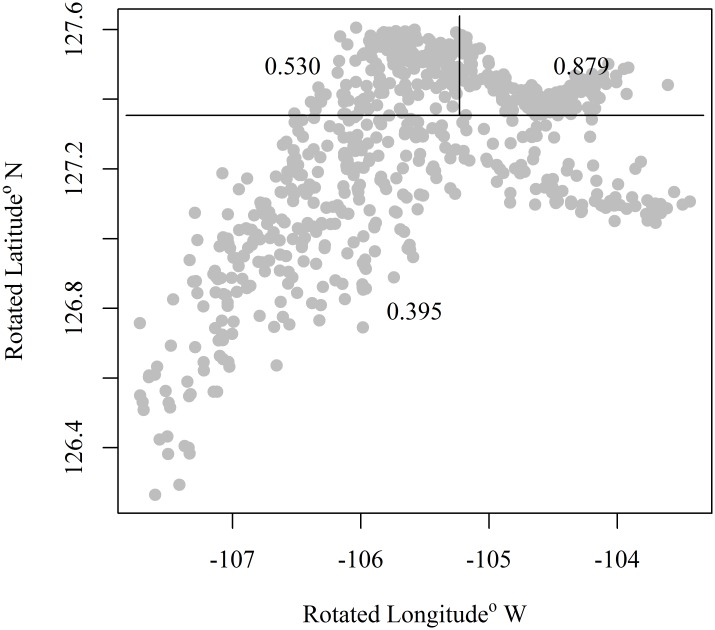
Rotated classification regression tree. Classification and regression tree model results for the proportion of female walleye pollock that are mature by location in the Gulf of Alaska for data rotated 30°clockwise. Numbers indicate the proportion mature in a given area.

Our analyses reveal a spatial pattern in the proportion of pollock mature at age or length in Shelikof Strait. The pattern is manifested as a gradient with a high proportion (>0.5) mature along the coast of the Alaska Peninsula to the northeast, and a low proportion mature (<0.4) to the southwest. A CART model divided the data space into five partitions explaining the geographic pattern in proportion mature. However, the CART model is constrained by a grid structure based on latitude and longitude, and produces breaks on a northeast-southwest diagonal within Shelikof Strait (Fig 4). To better model this pattern, the spatial coordinates were rotated 30° clockwise to correspond to axes that reflect the cross-strait and along-strait orientations, and the CART model was refit. This rotation reduced the number of breaks in the maturity data and thus the number of regions needed for estimating data weights (Fig 5). This approach to minimize the number of regions reduces the number of instances in which regions contain no trawl samples in a given year.

**Fig 6:**
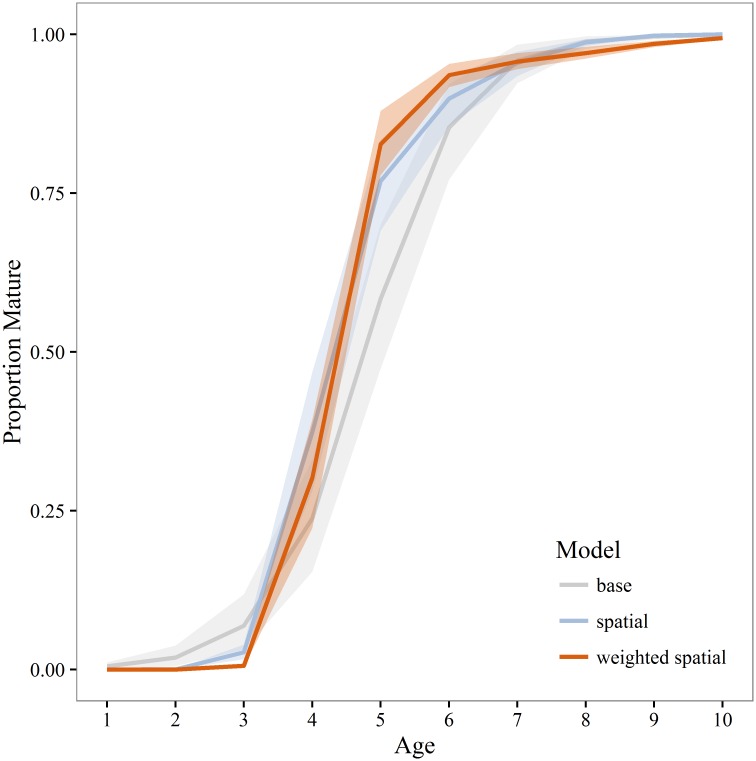
Walleye pollock maturity at age. Mean proportion mature at age for walleye pollock in the Gulf of Alaska during 2003–2013. Estimates represent the base, unweighted and weighted spatial models. Shaded areas are 95% bootstrap confidence intervals.

**Fig 7:**
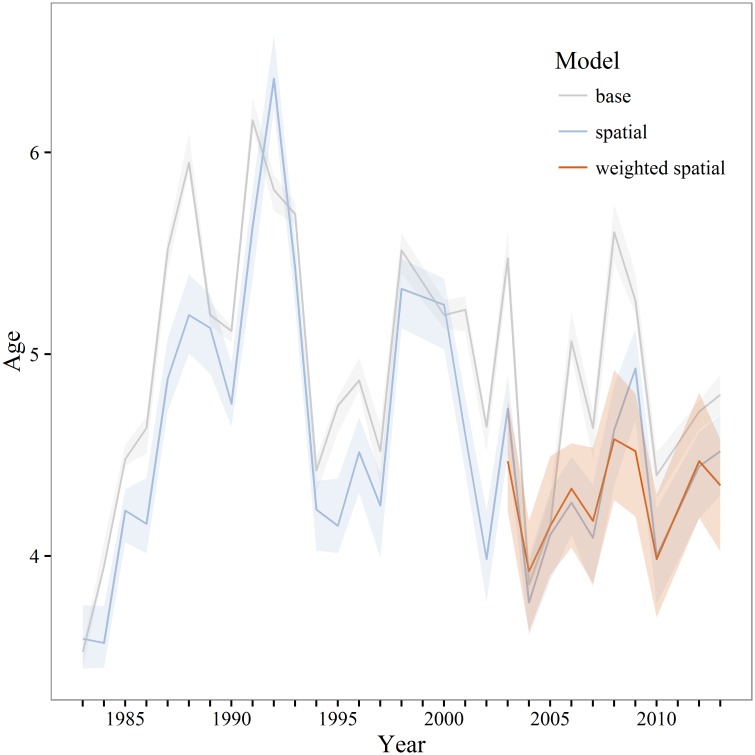
Walleye pollock A_50_. Estimates of 50% maturity at age (A_50_) for the base, unweighted and weighted spatial models. The base and unweighted spatial models are estimated from 1983–2013, the weighted spatial model is estimated from 2003–2013. Shaded areas are 95% model estimated confidence intervals.

**Fig 8:**
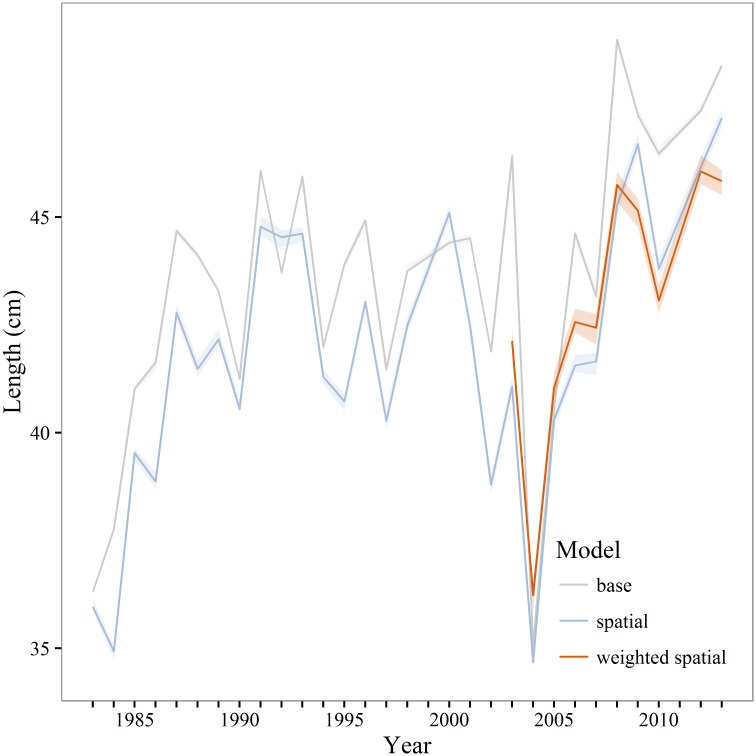
Walleye pollock *L*_50_. Estimates of 50% maturity at length (*L*_50_) for the base, unweighted and weighted spatial models. The base and unweighted spatial models are estimated from 1983–2013, the weighted spatial model is estimated from 2003–2013. Shaded areas are 95% model estimated confidence intervals.

**Fig 9:**
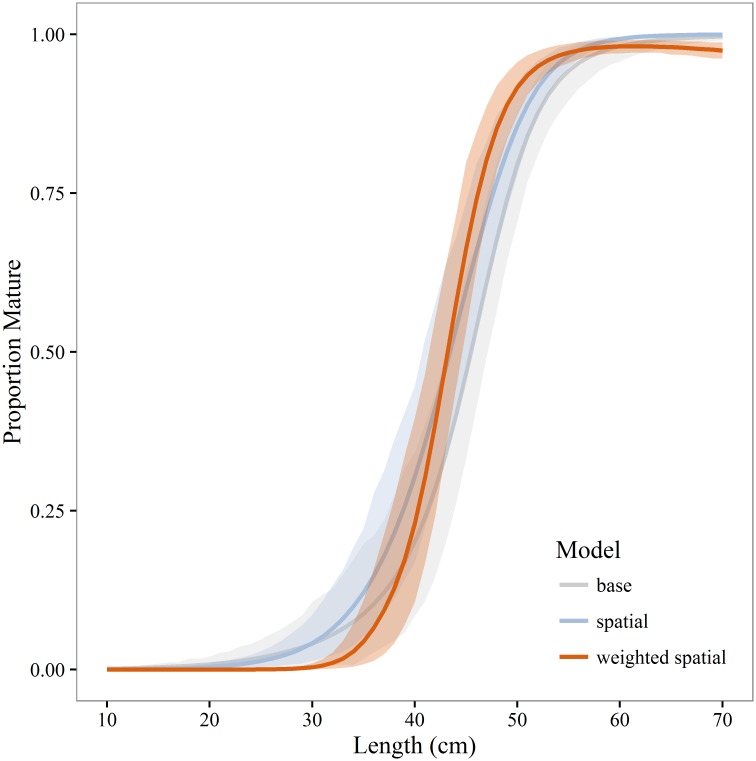
Walleye pollock maturity at length. Mean proportion mature at length for walleye pollock in the Gulf of Alaska during 2003–2013. Estimates represent the base, unweighted and weighted spatial models. Shaded areas are 95% bootstrap confidence intervals.

Annual estimates of maturity from the spatial and weighted spatial models were averaged by age for comparison to the maturity schedule used in the current stock assessment (base model). Compared to both the spatial and weighted spatial models the stock assessment maturity schedule overestimates the proportion of young (<age-4) mature fish and underestimates the proportion of mature fish from age-4 to age-7 (Fig 6). Differences between the unweighted and weighted spatial models were not statistically significant. In most years *A*_50_ and *L*_50_ was significantly lower for the spatial and weighted spatial models compared to the base model (Figs 7 and 8). There is a negative anomaly in all three estimates of *A*_50_ in 2004 (Fig 7) that is even more visible in estimates of *L*_50_ (Fig 8). The slope of the weighted spatial maturity at length (Fig 9) model is steeper than for the spatial and base models. The weighted spatial model significantly differs from the base model for fork lengths between 40–52 cm with a greater proportion of the population mature at a smaller size for the spatially-weighted model.

**Fig 10:**
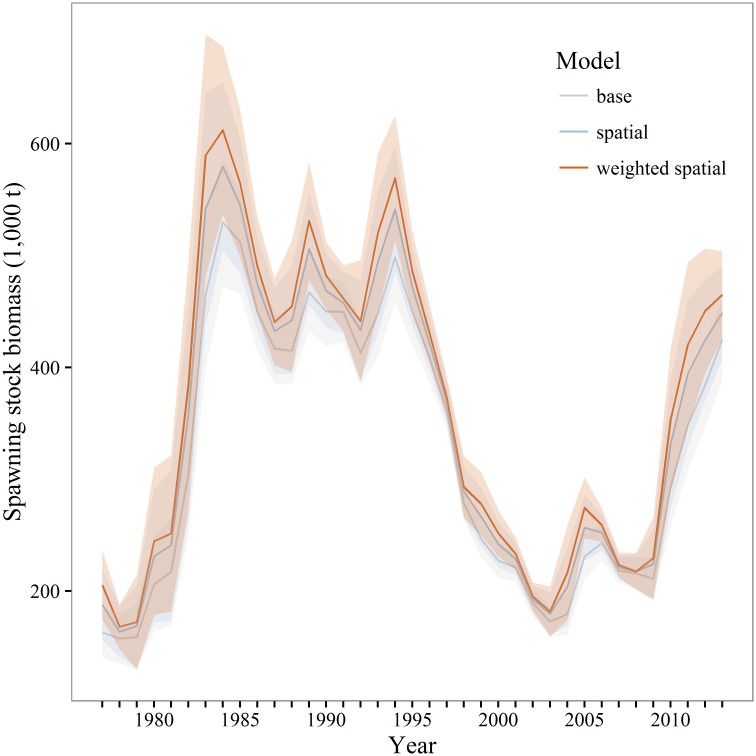
Walleye pollock spawning stock biomass. Estimates of walleye pollock spawning stock biomass (thousands of tons) for the Gulf of Alaska based upon age-averaged maturity estimates for 2003–2013.

**Fig 11:**
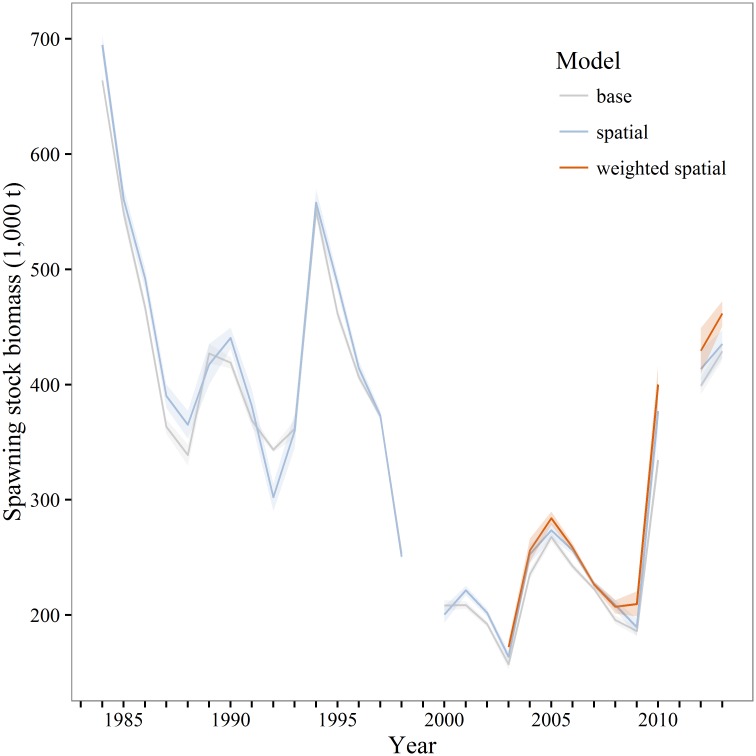
Spatially explicit walleye pollock spawning stock biomass. Estimates of walleye pollock spawning stock biomass (thousands of tons) for the Gulf of Alaska based upon annually varying maturity estimates. The base and unweighted spatial model are calculated for 1983–2013, the weighted spatial model is constrained by available abundance at location data and is calculated for 2003–2013.

Two sets of SSB estimates were used for evaluating the impacts of different models for estimating and incorporating maturity. The first is a comparison of SSB calculated from the mean maturity at age for all three models; this highlights differences between estimates using the methodology currently implemented in the stock assessment. The second is a comparison of SSB calculated from annually varying maturity estimates from all models. Estimates of SSB from the mean weighted and unweighted spatial models, based upon maturity in 2003–2013 though applied to all years (Fig 10), were always greater than the base model estimates of SSB (Table 4). When SSB is estimated on annually varying estimates of maturity, the weighted and unweighted spatial models were again greater in all years during 2003–2013. However, only the unweighted spatial model can be evaluated for earlier years as it does not utilize spatial biomass weights. A retrospective examination shows some years when the base model produces larger estimates of SSB than the unweighted spatial model (Fig 11).

**Table 4:** Differences between spatial or weighted spatial and base maturity model estimates of spawning stock biomass (1,000 t). Estimates of spawning stock biomass were estimated upon either mean or annually varying maturity estimates over 2003–2013.

	Mean maturity		Annual maturity	
SSB	Spatial	Weighted	Spatial	Weighted
mean	21.1	33.5	12.7	23.5
% diff	+7.6	+11.9	+4.7	+8.6
min	1.7	4.59	3.2	3.8
max	45.7	71.9	42.4	65.5
sd	15.6	25.3	11.5	17.1

## Discussion

Our analysis highlights the importance of considering the sampling scheme for collecting biological data used to estimate population parameters. We discovered prominent temporal and spatial patterns in maturity of walleye pollock in Shelikof Strait that may impact both our understanding of the biology and management of the species. There have been only a few attempts to evaluate latitudinal gradients in maturity in gadids [34–37] or other species [2, 38–40], examples of intra-stock spatial variability are rather limited [19, 39, 41]. For instance, both *L*_50_ and length at age of pollock tend to decrease with increasing latitude [19], consistent with observations of higher growth rates in the southern Bering Sea [42, 43]. In addition, interannual variability in *L*_50_ was inversely related to annual estimates of biomass of age 1+ pollock in the eastern Bering Sea, providing evidence of density dependent growth [19]. One confounding factor for developing maturity estimates that incorporate spatial information is the need to link disparate maturity and spatial abundance data. The approach that we outlined provides a method for identifying and incorporating observed spatial variability in a manner that is easily implemented and adaptive to changes in spatial structure (i.e., does not assuming fixed boundaries) and can be utilized for other species.

Potential reasons for spatial variability in maturity rates are wide ranging, and include temperature differences, changes in growth rates, population structure, migration or aggregation, and resource availability. Pollock in Shelikof Strait are considered a single genetic stock, inhabit a reasonably homogeneous environment (e.g., similar temperatures, salinities, and depths across the region), confounding our ability to uncover mechanisms responsible for the observed spatial trend in maturity. However, areas of pollock spawning locations were not found to be related to transport or temperature in Shelikof Strait [44]. Likewise, in the eastern Bering Sea, temperature does not appear to drastically change the spatial pattern of pollock spawning although seasonal warming coincides with the progression of the spawning season [45].

The spatial and weighted spatial model estimates of maturity indicate that fewer fish younger than age-4 are mature and more fish older than age-4 are mature when compared to the current maturity ogive used in annual stock assessments. When the maturity estimates are incorporated into stock assessment estimates of abundance there is a 4.7 to 11.9% difference increase in average SSB, depending on the maturity estimate used from 2003–2013. Using any estimate, the current strategy appears to be conservative under recent conditions, leading to a consistent underestimate of SSB for the time period evaluated. However, it is possible that this bias could be reversed in the future. Knowing that there is a spatial gradient in maturity a sampling design that works in conjunction with the “ground truth” hauls taken during hydroacoustic surveys may provide for more accurate estimates of maturity, or at least provide an indication of changing spatial biases. One such sampling design could be in the form of a limited number (6–8) of fixed trawl locations that are spatially separated and annually sampled in addition to the current sampling methodology.

As a caveat it should be noted that estimates of SSB in this study are based upon abundance for pollock in the whole of the GOA, not just Shelikof Strait. While the maturity estimates herein take into account spatial variability and relative abundance in Shelikof Strait, they do not account for possible spatial variability in the weight of pollock. This could be addressed by estimating SSB as the product of maturity at length and the spatial biomass at length, though this would not account for areas outside of Shelikof Strait. This method would, however, create a mismatch with a length-based estimate of maturity being incorporated into an age-structured stock assessment. This may well be a desirable objective particularly as length may be better associated with maturity as the process of maturation is likely to be driven by fish size as opposed to fish age, additionally length measurements are easier to obtain and are more precise than age estimates [2].

Another aspect that could influence the maturity estimates is the determination of female spatial abundance. This study assumes an even ratio of females to males, however trawl samples have been taken that are predominantly one sex. If sex ratios have strong spatial patterns the regional estimates of pollock maturity could be greatly influenced. Although lengths and ages are recorded by sex, methods have not yet been implemented for producing pollock biomass estimates by sex.

Our results are relevant to ecosystem-based fisheries management (EBFM), an approach that is broadly adopted by the North Pacific Fishery Management Council (NPFMC) for groundfish fishery management off Alaska [46]. For instance, pollock are important prey of Steller sea lions (*Eumetopias jubatus*), a large pinniped whose abundance west of Cape Suckling in the central Gulf of Alaska (144°W) declined severely in the 1970s to 1990s. Because of concerns that fishing on pre-spawning pollock could cause shifts in their spatial distribution and abundance that adversely affect sea lion foraging efficiency [43], the NPFMC implemented several precautionary measures, including area closures near sea lion rookeries and haulouts, as well as spatial and temporal apportionment of total allowable catches (TACs) into smaller sub-TACs to prevent localized prey depletions [46]. Further, a better understanding of biotic and abiotic factors affecting spatial and temporal patterns in maturity is consistent with EBFM, an approach that strives to balance diverse societal objectives by taking account of knowledge and uncertainties in biotic, abiotic and human components of ecosystems and their interactions. As marine ecosystems can exhibit complex behaviors, it is crucial for fishery managers to maintain resistance and resilience of exploited populations [44]. Thus, overfishing by highly size-selective fisheries are to be avoided, as such circumstances may lead to fishing-induced evolution of key biological traits, such as size of maturity; probabilistic maturation reaction norms may help detect such genetic effects [42]. However, the pollock fishery is managed with conservative harvest rates [10, 11] and interannual variability in *A*_50_ and *L*_50_ do not display directional trends that would be expected to arise from such genetic effects.

Although this study cannot address all of the components necessary for accurately determining maturity of GOA pollock, it clearly demonstrates that there is spatially explicit variability in maturity within Shelikof Strait. In this case, accounting for this variability increases the estimate of SSB. This has implications for estimates of stock productivity and therefore the harvest control rules used to manage this valuable fishery. Further it demonstrates a need for defined maturity sampling strategies that increase the ability to determine spatial and temporal trends in maturity and assure that catch specifications are determined upon the most accurate information possible, given the various constraints on resource assessment surveys. An important next step is to investigate ecological relationships between spatiotemporal variability in maturity and potential biotic and abiotic drivers. A better understanding of maturity trends and their relationships with ecological drivers could be incorporated into management strategy evaluations to evaluate management options for sustainable fisheries under climate change [47, 48].

## Supporting Information

S1 Dataset Walleye pollock maturity observations by length, weight, and age for the Gulf of Alaska. (ZIP)
